# Almond Shell-Derived Biochar for Lead Adsorption: Comparative Study of Pyrolysis Techniques and Sorption Capacities

**DOI:** 10.3390/molecules30204121

**Published:** 2025-10-17

**Authors:** Eva Pertile, Tomáš Dvorský, Vojtěch Václavík, Lucie Berkyová, Petr Balvín

**Affiliations:** Department of Environmental Engineering, Faculty of Mining and Geology, VSB—Technical University of Ostrava, 17. Listopadu 15/2172, 708 00 Ostrava, Czech Republic; eva.pertile@vsb.cz (E.P.); vojtech.vaclavik@vsb.cz (V.V.); lucie.berkyova.st@vsb.cz (L.B.); petr.balvin.st@vsb.cz (P.B.)

**Keywords:** biochar, microwave pyrolysis, slow pyrolysis, almond shells, lead adsorption, heavy metal removal, biomass waste, pH effect, adsorption isotherms

## Abstract

Lead (Pb(II)) contamination in water poses severe environmental and health risks due to its toxicity and persistence. This study compares almond shell-derived biochars produced by slow pyrolysis (SP) and microwave pyrolysis (MW), with and without KOH activation, focusing on structural properties and Pb(II) adsorption performance. Biochars were characterized by proximate and elemental analysis, BET surface area, FTIR spectroscopy, and adsorption experiments including pH dependence, kinetics, and equilibrium isotherms. Non-activated SP exhibited the highest surface area (S_BET_ = 693 m^2^·g^−1^), pronounced mesoporosity (≈73% of total pore volume), and the largest observed equilibrium capacities. KOH activation increased surface hydroxyl content but degraded textural properties; in MW samples, it induced severe pore collapse. Given the very fast uptake, kinetic modeling was treated cautiously: for non-activated biochars, Elovich adequately captured the time-course trend, whereas activated samples returned non-physical kinetic constants (e.g., negative k_2_) likely due to high post-adsorption pH (>11) and probable Pb(OH)_2_ precipitation. Equilibrium data (fitted over 50–500 mg·L^−1^) were better captured by the Freundlich and Redlich–Peterson models, indicating a mixed adsorption behaviour with contributions from heterogeneous site distribution and site-specific interactions. Optimal Pb(II) removal occurred at pH 4, with no measurable leaching from the biochar matrix. Overall, non-activated SP biochar is the most effective, sustainable and low-cost option among the tested materials for Pb(II) removal from water, avoiding aggressive chemical activation while maximizing adsorption performance.

## 1. Introduction

Lead (Pb(II)) contamination in aquatic environments remains a major environmental and public health concern due to its toxicity, persistence, and bioaccumulation [1]. Industrial activities such as smelting, battery manufacturing, mining, and waste mismanagement are the main sources of Pb(II) release into water bodies. Conventional methods for removing lead from aqueous solutions include chemical precipitation, ion exchange, membrane technologies, electrocoagulation, and reverse osmosis [2]. However, these approaches are often expensive, technologically demanding, and associated with the generation of secondary waste [3]. In this context, adsorption has emerged as an efficient and promising alternative due to its simplicity, low operational cost, and minimal technological requirements [4]. Particular attention has been devoted to adsorbent materials derived from renewable or waste resources, especially agricultural and food industry residues [5].

Biochar, a carbon-rich material produced by pyrolyzing biomass under limited oxygen supply, has shown great potential as an effective adsorbent for the removal of both metal and organic pollutants. This is attributed to its porous structure, high specific surface area, and the presence of functional groups (e.g., hydroxyl and carboxyl groups) [6].

Among various biomass sources, almond shells (*Prunus dulcis*), a common agricultural waste product in regions with intensive almond cultivation, have attracted attention due to their high lignin and cellulose content, low ash content, and good mechanical stability. These properties make them suitable for producing biochars with favorable physicochemical characteristics [5].

Pyrolysis conditions, including temperature, heating rate, and chemical activation, strongly influence the resulting material properties [7,8]. Slow pyrolysis (SP) typically yields high surface area and stable pore structure, whereas microwave pyrolysis (MW) offers rapid heating and energy efficiency [9]. Chemical activation, particularly with potassium hydroxide (KOH), further enhances the adsorption capacity of biochar by promoting microporosity, increasing surface area, and introducing oxygen-containing functional groups [10].

Despite growing interest in agro-waste-derived biochars, no previous study has systematically compared SP and MW biochars from almond shells (with and without KOH activation) for Pb(II) removal, while considering both textural properties and pH-induced artifacts in adsorption modelling. This study addresses this gap to identify the most effective, sustainable production route for water purification applications.

## 2. Results and Discussion

### 2.1. Characterization of Almond Shell-Derived Biochars

The experimental outcomes were significantly influenced by an inherent methodological difference between the two pyrolysis techniques employed. Specifically, microwave (MW) pyrolysis fundamentally operates through power input regulation rather than direct temperature control, resulting in thermal profiles that deviate substantially from those achieved during slow pyrolysis (SP). This fundamental distinction in heating mechanisms creates different reaction environments.

To address this methodological variance, our comparative analysis deliberately prioritized quantifiable material characteristics over nominal temperature parameters. We systematically evaluated specific surface area (BET), molecular structure (FTIR), thermal decomposition behavior (TGA), and standardized ASTM tests for biochar characterization. This comprehensive property-focused approach enabled meaningful scientific comparison between materials produced by these distinct pyrolytic pathways, effectively isolating the true effects of the heating mechanism from those of apparent temperature differences.

#### 2.1.1. Proximate and Elemental Analysis

The feedstock biomass of almond shells (AS-RAW) contained ~79% by weight of volatile sub-stances and ~3% by weight of ash, which corresponds to the literature data for lignocellulosic raw materials. After pyrolysis, the relative ash content in biochar decreased to ~2–5% by weight due to the removal of organic substances to volatile and condensable fractions.

For the purpose of mutual comparability, all results were recalculated on a dry matter basis, i.e., with respect to the removal of moisture. Thus, the elemental composition values (C, H, N, S) in Table 1 are expressed on a dry basis for biochar samples, while the AS-RAW values were originally measured on an as-received basis and subsequently converted to dry matter for consistent comparison. Data with moisture content can be found in Supplementary Information Table S1.

The elemental composition shows that AS-RAW contains ~45.6 wt.% C, 6.4 wt.% H, 1.74 wt.% N, and 0.3 wt.% S. Thermal processing markedly increased carbon content in AS (87.86 wt.%) and AS-RAW-MW (87.93 wt.%), whereas KOH activation reduced the carbon content to 76.56 wt.% (AS-KOH-SP) and 76.13 wt.% (AS-KOH-MW). Concurrently, hydrogen and nitrogen contents decreased in all thermally processed samples.

#### 2.1.2. Thermal Decomposition and Product Distribution

**Figure 1:**
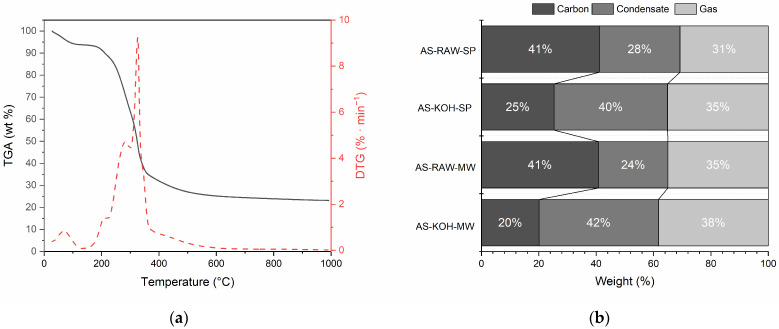
DTG curve of raw almond shells (**a**) and product distribution from different pyrolysis/activation routes (**b**) Product distribution from different pyrolysis/activation routes. “Liquid” denotes the total condensate (aqueous pyrolysis water + organic fraction) collected gravimetrically; phases were not separated.

The thermal decomposition profile of almond shells under nitrogen (Figure 1a) revealed an initial weight loss at 50–100 °C due to moisture removal, followed by major degradation between 200 and 400 °C, with a DTG peak at ~320 °C. This stage corresponds to hemicellulose (200–250 °C) and cellulose (300–350 °C) degradation [11]. Above 370 °C, slow decomposition of lignin and more complex structures occurred.

Product distribution analysis showed that slow pyrolysis preserved the highest char yield, while KOH activation decreased char yield and increased condensate and gas fractions. Microwave processing promoted gas formation, while KOH + MW processing yielded the highest total liquid and gas products but the lowest char yield. KOH pretreatment promoted dehydration and cracking reactions, leading to higher yields of condensable oxygenates and pyrolysis water, which explains the observed increase in the “liquid” fraction (Figure 1b).

#### 2.1.3. Textural Properties

Table 2 summarizes BET surface area and pore characteristics. AS-RAW-SP exhibited the highest surface area (693 m^2^·g^−1^) and total pore volume (0.598 cm^3^·g^−1^), dominated by mesopores (~73%). KOH activation significantly reduced S_BET_ to 476 m^2^·g^−1^ and V_meso_ to 0.065 cm^3^·g^−1^. Microwave processing caused a more pronounced reduction in surface area (147 m^2^·g^−1^), and the KOH-MW combination led to the lowest values (44.6 m^2^·g^−1^; 0.050 cm^3^·g^−1^ total pore volume).

**Figure 2:**
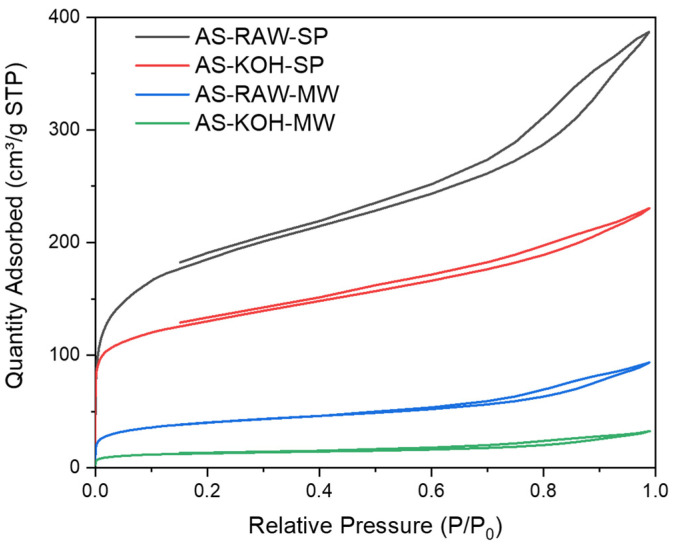
N_2_ adsorption–desorption isotherms (AS-RAW-SP, AS-RAW-MW, AS-KOH-SP, AS-KOH-MW).

Figure 2 shows N_2_ adsorption–desorption isotherms. AS-RAW-SP displayed type IV isotherms with H3 hysteresis loops, typical for mesoporous materials, while KOH activation preserved the isotherm shape but reduced capacity. MW-treated samples exhibited significantly lower uptake, particularly AS-KOH-MW (<30 cm^3^/g STP). These trends align with literature observations [12].

#### 2.1.4. FTIR Spectral Analysis

**Figure 3:**
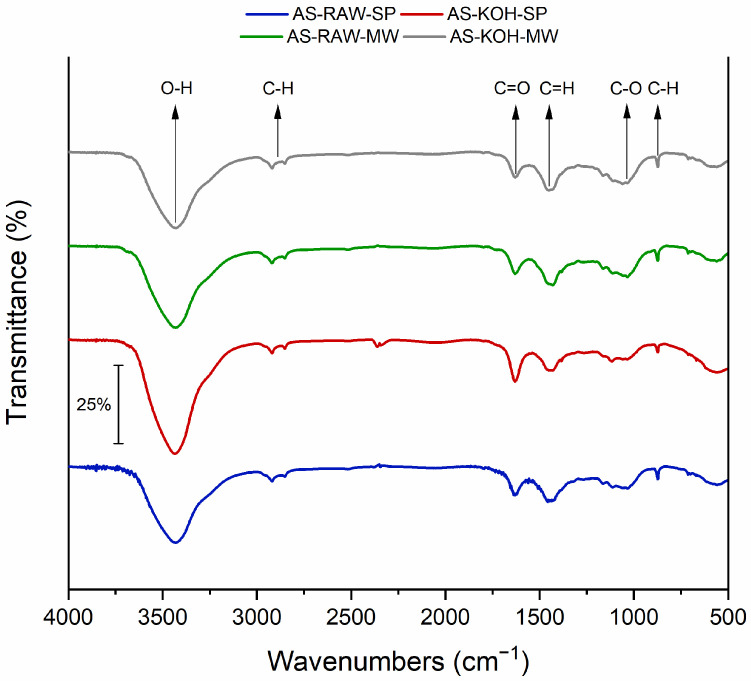
FTIR spectra of almond shell-derived biochars (KBr pellet). Assignments indicated.

FTIR spectra (Figure 3) revealed characteristic bands consistent with lignocellulosic biomass-derived carbons. A broad band at ~3400 cm^−1^ corresponds to O–H stretching vibrations from hydroxyl groups in phenolic, alcoholic, and carboxylic functionalities [13]. The intensity of this band was highest in KOH-activated samples, suggesting increased hydroxyl content due to ester bond hydrolysis during alkaline treatment. Bands at ~2920 and ~2850 cm^−1^ were assigned to C–H stretching in aliphatic –CH_2_ and –CH_3_ groups, associated with lignin-derived structures [14]. A peak near 1700 cm^−1^ corresponded to C=O stretching in carbonyl groups (esters, ketones, and carboxylic acids) [15]. This band was diminished in KOH-treated samples, indicating cleavage of ester/amide linkages. The region 1000–1500 cm^−1^ featured bands from C–O stretching and C–H bending, associated with polysaccharides, phenols, and alcohols [16]. The most significant alterations occurred in AS-KOH-MW, consistent with its minimal surface area and pore volume.

#### 2.1.5. Physico-Chemical Differences and Implications

Biochars produced via slow pyrolysis exhibited the highest porosity and specific surface area, making them particularly suitable for the adsorption of heavy metals and organic pollutants. This is attributed to the gradual thermal decomposition and uniform carbonization, which promote the development of a stable and hierarchical pore structure [17].

In contrast, microwave pyrolysis resulted in reduced textural parameters due to rapid and uneven heating, which can lead to pore collapse and lower surface area. Although microwave-assisted pyrolysis is more energy-efficient and faster, its effectiveness in producing adsorbents depends heavily on process optimization, such as temperature and residence time [18].

KOH activation increased the abundance of surface hydroxyl groups, enhancing the chemical affinity for metal ions. However, when combined with microwave pyrolysis, it may lead to excessive structural degradation and pore blockage, thereby reducing the overall adsorption capacity [19].

From an application perspective, slow pyrolysis without chemical activation appears optimal for adsorption-based remediation due to its favorable balance of surface area, porosity, and functional group availability. Conversely, KOH + microwave treatment may be more suitable for energy-oriented applications, such as bio-oil or syngas production, where higher liquid and gas yields are desired [17].

### 2.2. Adsorption Kinetics

**Figure 4:**
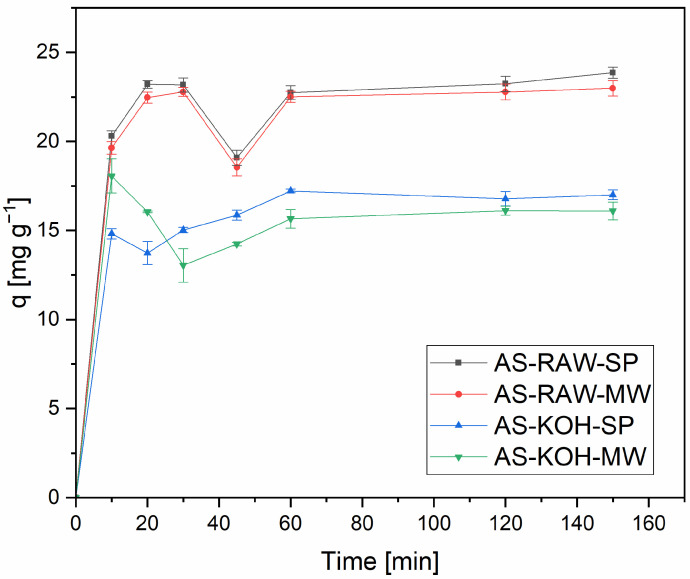
Adsorption kinetics: Pb(II) removal (%) vs. contact time for all biochars. Conditions: adsorbent dose = 0.1 g in 50 mL; initial concentrations 50 mg L^−1^; contact time = 10, 20, 30, 45, 60, 120, and 150 min; T = 22 ± 2 °C; shaker = 180 rpm. Error bars represent the standard deviation (SD) of three independent adsorption experiments. Complete triplicate data (q_1_–q_3_, mean, SD, RSD) are provided in Table S3 (Supplementary Information).

The kinetics of Pb(II) adsorption onto biochars prepared via slow pyrolysis (SP) and microwave pyrolysis (MW) were evaluated using several commonly applied models (PFO, PSO, Elovich, IPD). Adsorption experiments were conducted using 0.1 g of biochar in 50 mL of Pb(II) solution (initial concentration of Pb(II): 50 mg·L^−1^). In the main text, given the very rapid uptake, we summarize empirical descriptors of practical relevance (e.g., t_90_) and do not interpret model parameters mechanistically. Adsorption progress for all biochars is shown in Figure 4. The complete fits of all tested models are provided in the Supplementary Information (Figures S3–S6).

Although adsorption kinetics can be fitted by various models (see Supplementary Materials: Figures S3–S6), Pb(II) uptake reached near-complete values within minutes (typically t_90_ ≈ 5–10 min). Under such conditions, most time points beyond the initial few minutes carry limited kinetic information [20], and model regressions may become ill-conditioned, occasionally yielding non-physical constants (e.g., unrealistically high rate constants or negative k_2_). Therefore, we refrain from mechanistic interpretation of kinetic parameters in the main text and rely on empirical descriptors (t_90_, q_e_) to compare practical performance.

Subsequent equilibrium and pH-effect experiments were conducted with non-activated biochars (AS-RAW-SP, AS-RAW-MW), which provided higher Pb(II) uptake. For KOH-activated materials, post-adsorption pH frequently increased up to ~11, where hydrolysis/precipitation of Pb(OH)_2_ may occur; such pH-induced artefacts plausibly contribute to inconsistencies in fitted kinetic parameters and lower apparent removal efficiency [21,22,23]. In summary, due to very fast equilibration, kinetic modeling in the main text is intentionally limited to descriptive use; detailed trial fits are retained in the Supplementary Materials for transparency.

### 2.3. Effect of pH

Solution pH plays a critical role in both the efficiency of Pb(II) removal and the reliability of kinetic modeling. While Pb(II) adsorption generally increases with rising pH due to reduced competition from H^+^ ions, precipitation of Pb(OH)_2_ above pH 6 can artificially enhance apparent removal efficiencies. For KOH-activated biochars, the post-adsorption pH exceeded 11 after 150 min, well above the precipitation threshold. Under these highly alkaline conditions, Pb(II) removal was likely dominated by precipitation rather than surface adsorption, resulting in poor fits to kinetic models and non-physical parameters (e.g., negative k_2_ values). Consequently, KOH-activated biochars were excluded from further adsorption studies.

Subsequent investigations of pH effects and adsorption isotherms were therefore conducted exclusively with non-activated biochars, which maintained final pH values between 7 and 8. This ensured that surface adsorption remained the primary removal mechanism, allowing for consistent kinetic behavior. The highest Pb(II) uptake was observed at pH 4, a condition that minimized precipitation while promoting favorable electrostatic interactions between Pb(II) ions and surface functional groups.

**Figure 5:**
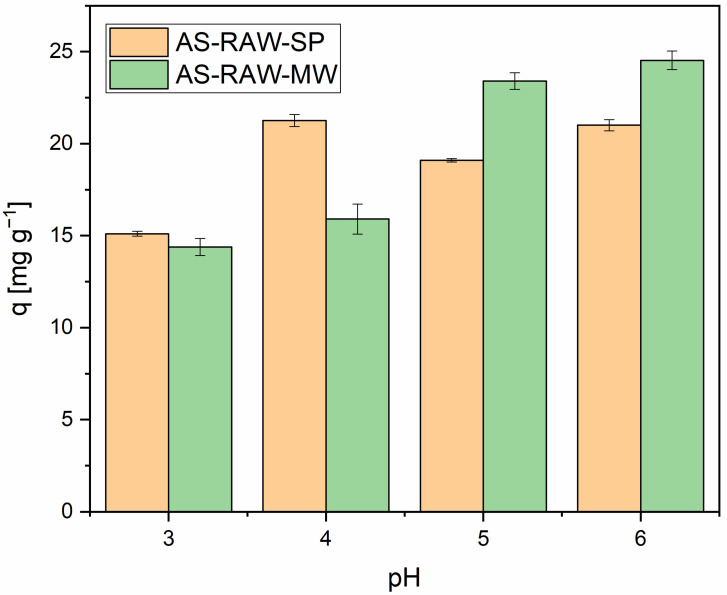
Effect of pH on Pb(II) adsorption. Conditions: adsorbent dose = 0.1 g in 50 mL; 50 mg L^−1^; pH = 3–6 (adjusted with 1 M HNO_3_/NaOH); T = 22 ± 2 °C; shaker = 180 rpm; *n* = 3 (mean).

Methodologically, avoiding artifacts from excessive alkalinity is essential for accurate evaluation of adsorbent performance, particularly when comparing materials with different surface chemistries. Maximum Pb(II) adsorption due solely to surface interactions was observed at pH 4–5 (Figure 5), consistent with previous reports [22,24,25].

From an environmental perspective, understanding the pH-dependent behavior of Pb(II) adsorption is crucial for designing effective biochar-based remediation strategies, particularly in acidic mine drainage or industrial effluents where pH control is challenging and metal speciation is highly dynamic. Based on pH-dependent adsorption experiments conducted in the range of pH 3–6, pH 4 was selected for subsequent tests. This condition ensured near-complete Pb(II) removal while remaining representative of realistic industrial wastewater, thereby providing both optimal uptake and methodological clarity by avoiding pH-induced precipitation artifacts.

### 2.4. Adsorption Isotherms

**Figure 6:**
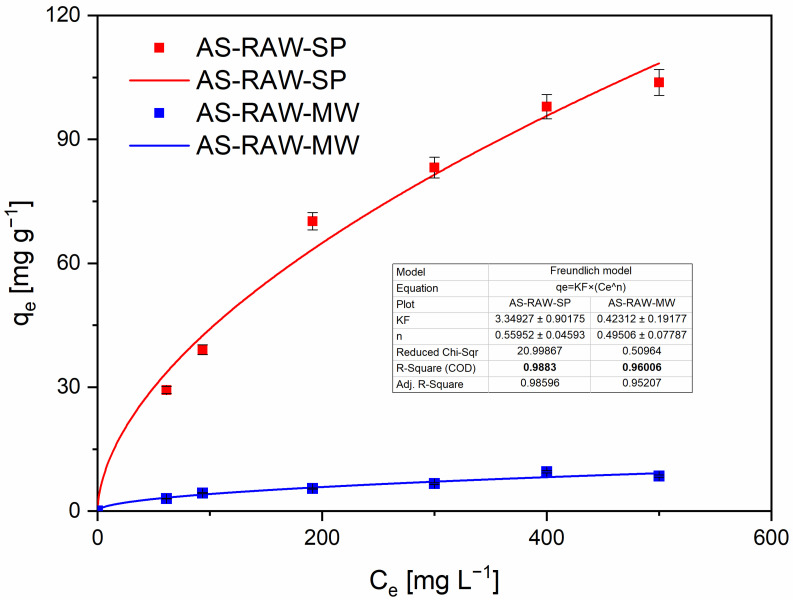
Freundlich adsorption isotherm for Pb(II) removal by almond-shell biochars prepared by slow pyrolysis (AS-RAW-SP) and microwave pyrolysis (AS-RAW-MW). Experimental data points and nonlinear model fits are shown; fitted parameters are given within the figure. Conditions: adsorbent dose = 0.10 g in 50 mL; initial Pb(II) concentrations 50–500 mg·L^−1^; equilibrium pH = 4 (adjusted with HNO_3_/NaOH); contact time = 60 min; T = 22 ± 2 °C; shaker = 180 rpm. Fit method: nonlinear regression (OriginPro 2023). The experimental value at 600 mg·L^−1^ is retained in Table S4 (Supplementary Materials) for completeness but was excluded from regression.

**Figure 7:**
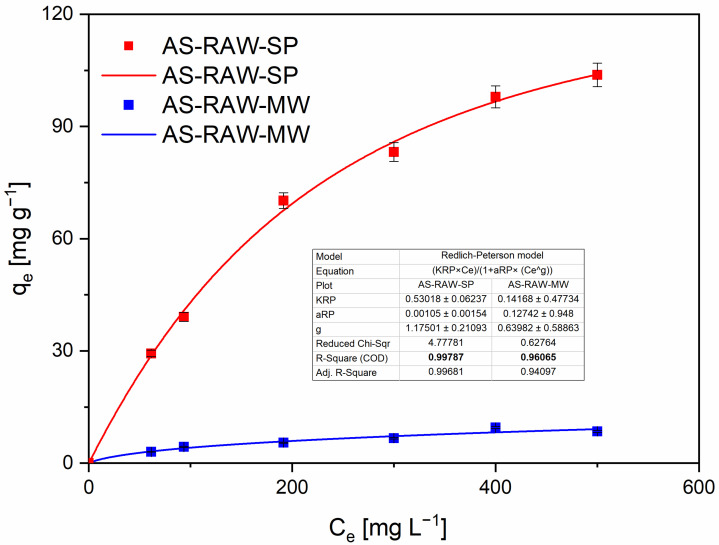
Redlich–Peterson adsorption isotherm for Pb(II) removal by almond shell biochars prepared by slow pyrolysis (AS-RAW-SP) and microwave pyrolysis (AS-RAW-MW). Experimental data points and model fits are shown; fitting parameters are provided within the figure. Conditions: adsorbent dose = 0.1 g in 50 mL, initial Pb(II) concentrations 50–500 mg·L^−1^, equilibrium pH = 4 (adjusted with HNO_3_/NaOH), contact time = 60 min, T = 22 ± 2 °C, shaker = 180 rpm. Fit method: nonlinear regression (OriginPro 2023). The experimental value at 600 mg·L^−1^ is retained in Table S4 (Supplementary Materials) for completeness but was excluded from regression.

Within the concentration range tested, the equilibrium data were better captured by the Freundlich and Redlich–Peterson (R–P) models than by Langmuir or Dubinin–Radushkevich (D–R), as indicated by goodness-of-fit metrics (Figure 6 and Figure 7). Langmuir and D–R fits are therefore reported only in the Supplementary Information for completeness (Figures S7 and S8). Despite an acceptable R^2^, the D–R fit yielded parameter values of questionable physical plausibility (e.g., unusually large k_DR_), producing non-informative capacity estimates; accordingly, D–R-derived q_max_ is not interpreted in the main text. 

The R–P model provided the best overall description of the isotherms and supports a mixed adsorption mechanism combining features of both homogeneous and heterogeneous adsorption—a conclusion that is consistent with the literature on heavy-metal sorption by biochars [26]. Isotherm experiments were performed up to an initial Pb(II) concentration of 600 mg·L^−1^ (see Methods). The experimental value measured at 600 mg·L^−1^ deviated from the trend observed at lower concentrations, which likely reflects the onset of surface saturation and reduced accessibility of active sites. For this reason the 600 mg·L^−1^ point was excluded from the nonlinear regressions; the raw value is reported in Table S4 (Supplementary Materials). All model fits presented in the main text were therefore performed using the concentration range 50–500 mg·L^−1^. Instead, we report the experimentally observed maximum adsorption capacity: q_e_ = 103 ± 2.5 mg·g^−1^ (measured at C_0_ = 500 mg L^−1^).

AS-RAW-SP exhibited a significantly higher adsorption capacity than AS-RAW-MW. This superior performance can be attributed to differences in pyrolysis dynamics: slow pyrolysis promoted the development of a well-connected mesoporous network (S_BET_ = 693 m^2^·g^−1^, V_meso_ = 0.437 cm^3^·g^−1^), whereas microwave pyrolysis yielded a lower surface area and pore volume (S_BET_ = 147 m^2^·g^−1^, V_meso_ = 0.107 cm^3^·g^−1^). In addition, FTIR spectra revealed a higher intensity of O–H and C=O bands in AS-RAW-SP, indicating a greater abundance of polar functional groups that enhance Pb(II) coordination.

Overall, the isotherm analysis confirms that adsorption performance is not governed by a single parameter (e.g., surface area) but rather by the combined effects of pore structure and surface chemistry.

### 2.5. Linking Biochar Properties to Adsorption Behavior

The adsorption performance of the almond shell-derived biochars can be directly related to their physicochemical characteristics, including proximate and elemental composition, BET surface area, pore structure, and FTIR spectra.

The biochar produced via slow pyrolysis (AS-RAW-SP) exhibited the highest BET surface area (693 m^2^·g^−1^) and a predominantly mesoporous structure, with mesopores accounting for approximately 73% of the total pore volume. This highly accessible surface area and hierarchical pore architecture facilitate the diffusion and binding of Pb(II) ions, resulting in the highest adsorption capacities observed in the isotherm experiments. Similar correlations between mesoporosity and enhanced heavy metal adsorption have been reported for lignocellulosic biochars [27].

In contrast, KOH activation unexpectedly reduced the surface area of slow-pyrolyzed biochar to 476 m^2^·g^−1^, likely due to partial pore collapse or blockage during the chemical treatment [17]. Nevertheless, FTIR analysis revealed an increased abundance of surface hydroxyl (O–H) groups after KOH activation [28], which can serve as active sites for Pb(II) complexation [19]. This suggests that although the textural properties were diminished, the surface chemistry was enhanced, potentially compensating for the loss of surface area in terms of metal ion affinity. Similar trends have been observed for alkali-treated agricultural residues [19].

Microwave pyrolysis (AS-RAW-MW) resulted in a sharp decrease in surface area (147 m^2^·g^−1^) and total pore volume, attributed to the rapid and uneven heating that can lead to pore structure collapse [18]. This was reflected in a reduced Pb(II) adsorption capacity. The combined KOH and microwave treatment (AS-KOH-MW) further reduced the surface area to just 44.6 m^2^·g^−1^ and yielded the lowest adsorption performance, suggesting that excessive activation under rapid heating conditions may severely impair the pore network [29], even in the presence of surface functional groups [30].

FTIR analysis also revealed that KOH activation led to a reduction in carbonyl (C=O) band intensity, consistent with hydrolysis of ester or amide linkages [31], and an increase in hydroxyl group content [32], which may promote metal ion binding via ion exchange or surface complexation. Microwave treatment caused modifications in aliphatic C–H bands [33], suggesting rearrangements in the carbon matrix that could influence non-specific adsorption interactions.

Overall, the results highlight that optimal Pb(II) adsorption is not solely governed by surface area but rather arises from a balance between textural properties (pore volume, surface area, pore size distribution) and surface chemistry (type and abundance of functional groups). For almond shell-derived biochars, slow pyrolysis without chemical activation provided the most favorable combination of these factors, explaining its superior performance in the adsorption experiments.

For context, Table S2 in the Supplementary Information summarizes reported Pb(II) adsorption capacities of representative agro-waste biochars under comparable experimental conditions. Our non-activated slow-pyrolyzed almond shell biochar performs in the upper range of these reported values, while maintaining a simple preparation route and controlled pH conditions (see Table S2, Supplementary Materials). This comparison highlights that our material performs competitively within the literature while maintaining simplicity of preparation and controlled pH without chemical activation.

## 3. Materials and Methods

### 3.1. Biomass Preparation

Almond shells (denoted as AS), used as the initial biomass for adsorbent preparation, were obtained from commercially available almonds with no information on the country of origin. To preserve the natural character of the material and considering potential industrial-scale applications, the AS were not washed prior to further processing.

The biomass was mechanically milled using a TESTCHEM—LMN-180 knife mill (Testchem, Radlin, Poland) with a 5.0 mm sieve. It was then dried at 105 °C to constant weight using a UF 260 drying oven (Memmert, Schwabach, Germany). Sieving was performed using a Retsch AS200 oscillating sieve shaker (Retsch GmbH, Düsseldorf, Germany) with Retsch sieves, and the 3.0–4.0 mm fraction was selected.

### 3.2. Chemical Activation of Biomass

Following mechanical treatment, AS samples were prepared for thermal and microwave pyrolysis. Chemical activation was carried out using potassium hydroxide solution (KOH, p.a., Penta s.r.o., Prague, Czech Republic). Two activation variants were applied: without KOH (denoted as AS-RAW) and with the addition of KOH at a solid: KOH weight ratio of 1:1 (denoted as AS-KOH). A total of 100 g of AS biomass was combined with either 0 or 100 g of KOH and 100 mL of demineralized water. The mixture was stirred for 8 h at room temperature, then dried at 105 ± 1 °C to constant weight. KOH activation is known to increase microporosity and specific surface area, thereby enhancing the adsorption capacity of biochar [10,34].

Following activation, the samples were thoroughly washed according to the procedure of [35]. In brief, the chars were rinsed with hot deionized water until the filtrate reached neutral pH and subsequently dried at 105 °C to constant mass. This step removes residual KOH and yields biochars suitable for further characterization.

### 3.3. Pyrolysis Processes

Microwave pyrolysis was carried out in a sealed apparatus equipped with a 500 mL quartz reactor containing 50 g of activated or non-activated biomass and 5 g of pre-prepared biochar (denoted AS-P). This auxiliary (or “starter”) biochar, produced by thermal pyrolysis at 600 °C (heating rate 10 °C·min^−1^, 1 h, N_2_ atmosphere), served as a microwave absorber–facilitating the initial heating and ensuring more uniform energy distribution, thus reducing the risk of sparking and uneven biomass decomposition [36].

Prior to microwave heating, the system (Supplementary Figure S1) was purged with nitrogen for 5 min (3 L·min^−1^) to establish an inert atmosphere. Microwave pyrolysis was performed at 400 W for 30 min. Note that MW conditions are reported as applied microwave power rather than controlled reactor temperature. After completion, the biochar was cooled under a nitrogen stream to room temperature. The selected conditions were based on protocols described by Vaštyl et al. (2022) and Jankovská et al. (2024) [36,37].

Slow pyrolysis was conducted under comparable conditions to those used in microwave pyrolysis. The same parameters were maintained: biomass mass (50 g), reactor volume (500 mL), nitrogen flow (3 L·min^−1^), and a cooling and condensation system. The temperature ramp was set to 10 °C·min^−1^ with a final temperature of 600 °C and a residence time of 1 h. The process was carried out under an inert atmosphere, and the resulting material was cooled under nitrogen to room temperature. These conditions followed the methodology of Grycová et al. (2018) [10]. A schematic of the slow pyrolysis setup is provided in Supplementary Figure S2.

Slow pyrolysis (SP) was temperature-programmed (final t = 600 °C), whereas microwave pyrolysis (MW) was operated under power control (400 W), leading to different apparent temperature regimes. Full operating parameters are provided in the Supplementary Materials.

#### Post-Pyrolysis Biochar Treatment

Each sample was first washed with 1 L of 0.15 M HCl solution, followed by two rinses with boiling demineralized water (1 L), and finally several rinses with cold demineralized water until neutral pH was achieved (typically after five washes). Filtration was carried out using a Milipore WP vacuum pump and a Büchner funnel with No. 3 filter paper. The washed material was dried at 105 °C to constant weight and stored in a desiccator.

The final product was sieved through standard laboratory sieves to obtain a particle size fraction < 0.1 mm.

### 3.4. Biochar Characterization

A comprehensive characterization of biochar was conducted using a broad range of analytical techniques, providing an integrated overview of its physicochemical properties. To gain a detailed understanding of the material’s structure and composition, a systematic approach incorporating complementary methods was employed.

Proximate analysis was used to determine fundamental parameters, including moisture content, volatile matter, fixed carbon, and ash. Elemental analysis (CHNS) enabled precise quantification of carbon, hydrogen, nitrogen, and sulphur. Thermogravimetric analysis (TGA) was applied to monitor weight changes in the raw sample as a function of temperature, offering insights into its thermal stability relevant to pyrolysis processes.

Physisorption measurements characterized surface area and pore structure, which are critical for evaluating the adsorption capacity of biochar. Fourier-transform infrared spectroscopy (FTIR) was employed to identify functional groups.

Proximate analysis (moisture, volatile matter, ash, fixed carbon) was carried out using a LECO TGA 701 (LECO Corporation, St. Joseph, MI, USA) following ASTM D7582 [38]. Moisture content was measured as mass loss at 105 °C to constant mass. Proximate values for raw almond shells are reported on an as-received basis (i.e., including natural moisture), while values for biochars are reported on a dry basis. CHNS elemental analysis was performed on a LECO CNS628 analyzer (LECO Corporation, St. Joseph, MI, USA) (dry basis). Representative instrumental parameters and replicate raw data are provided in the Supplementary Information, while full protocols are available from the laboratory upon request.

Values in Table 1 are reported on a dry-weight basis (moisture = 0). Original as-received moisture contents and the raw proximate data are available in Supplementary Materials Table S1. Where necessary, numbers in the text have been recalculated to a dry basis and this is stated explicitly.

### 3.5. Batch Adsorption Experiments

To assess the adsorption performance of AS-RAW and AS-KOH biochars, a series of batch experiments were conducted, applying mathematical models to describe both adsorption kinetics and equilibrium behaviour. The experimental design aimed to characterize adsorption over time, evaluate the influence of pH, and determine equilibrium parameters.

All adsorption tests were carried out using 0.1 g of biochar and 50 mL of Pb(II) solution in 100 mL polyethylene centrifuge tubes, resulting in an initial concentration of 50 mg·L^−1^, isotherms used C_0_ = 50–500 mg·L^−1^. The stock Pb(II) solution was prepared from Pb(NO_3_)_2_, and intermediate concentrations were obtained by dilution with distilled water. For isotherm experiments, the pH was adjusted to 4 (HNO_3_/NaOH) and maintained throughout equilibration, as selected from the pH-dependent adsorption tests (Section 2.3).

Adsorption kinetics were investigated using contact times of 10, 20, 30, 45, 60, 120, and 150 min. To study the effect of solution pH on Pb(II) adsorption, the pH of the solutions was adjusted within the range of 3–6 using 1 M HNO_3_ and NaOH and measured with a Hanna EDGE pH meter (Hanna Instruments Czech s.r.o., Prague, Czech Republic). Equilibrium adsorption experiments were performed at initial Pb(II) concentrations of 50, 100, 200, 300, 400, 500, and 500 mg·L^−1^. An additional point at 600 mg·L^−1^ was measured but excluded from model fitting (raw value in Table S4, Supplementary Materials). All reagents used were of analytical grade.

To ensure the reliability of the adsorption data, control experiments without biochar were conducted under identical conditions to exclude the possibility of Pb(II) release from the experimental setup or materials. Additionally, the pH of each solution was measured both before and after the adsorption process. This allowed monitoring of pH shifts induced by the biochar, which were particularly pronounced in KOH-activated samples, where final pH values often exceeded 11. Such alkaline conditions may interfere with adsorption by promoting Pb(OH)_2_ precipitation, thus affecting the interpretation of removal efficiency. Experiments were conducted at ambient laboratory temperature using an IKA Incubator Shaker KS 4000i (IKA-Werke GmbH & Co. KG, Staufen, Germany) Control set to 180 rpm.

#### Kinetic and Equilibrium Models

To gain a comprehensive understanding of Pb(II) adsorption mechanisms on biochar, various mathematical models were applied to describe both kinetic and equilibrium aspects of the process. Kinetic studies, which provide insights into the rate and controlling mechanisms of adsorption, were evaluated using three commonly employed models: pseudo-first-order (PFO), pseudo-second-order (PSO), the Elovich model and intraparticle diffusion (IPD). These models help distinguish between physical adsorption and chemisorption and identify rate-limiting steps.

Equilibrium adsorption data were analysed using four theoretical isotherm models: the Langmuir isotherm, which assumes monolayer adsorption on a homogeneous surface; the Freundlich isotherm, suitable for multilayer adsorption on heterogeneous surfaces; the Redlich–Peterson isotherm, which combines features of both Langmuir and Freundlich models; and the Dubinin–Radushkevich isotherm, which characterizes adsorption on microporous materials with a uniform distribution of activation energies.

Detailed mathematical expressions of all kinetic and isotherm models, including definitions of parameters and constants, are provided in the Supplementary Information (Supplementary Materials: Kinetic and Equilibrium Models).

### 3.6. Methodology for the Determination of Pb

Lead concentrations were determined by atomic absorption spectrometry (AAS) using a contrAA^®^ 700 spectrometer (Analytik Jena, Jena, Germany) operated in flame mode (acetylene–air or acetylene–nitrous oxide, as appropriate). Measurements followed the accredited procedure “Determination of Selected Metals by Flame AAS in Aqueous Extracts and Solid Materials” as implemented at the certified laboratories of IET CEET, VSB–Technical University of Ostrava. All measurements were performed in triplicate and reported values are arithmetic means of three independent determinations. The analytical precision of the routine flame AAS procedure in our laboratory is typically ≈3% RSD.

Model solutions of Pb^2+^ were prepared by serial dilution of a stock solution with a concentration of 1 g·L^−1^, using redistilled water as the solvent. For each experiment, both the initial and final concentrations of Pb(II) (after adsorption) were measured to evaluate adsorption efficiency.

## 4. Conclusions

This study demonstrated that almond shell-derived biochars produced via slow pyrolysis exhibit superior physicochemical properties and lead adsorption performance compared to those produced via microwave pyrolysis. The slow pyrolysis biochar (AS-RAW-SP) showed the highest BET surface area and mesoporosity. In contrast, microwave pyrolysis resulted in reduced surface area and pore volume, leading to lower adsorption efficiency. Chemical activation with KOH increased the abundance of surface hydroxyl groups but simultaneously caused structural degradation, particularly under microwave conditions, which negatively affected adsorption behavior. Given the near-instantaneous uptake (minutes), detailed kinetic modeling was not considered mechanistically informative in the main text; we therefore focused on empirical descriptors of practical performance and provide trial fits only in the Supplementary Information. The pH-dependent experiments confirmed that optimal lead removal occurred at pH 4, minimizing precipitation artifacts and maximizing adsorption efficiency. Control experiments verified the absence of Pb(II) release from the biochar matrix. Overall, slow pyrolysis without chemical activation proved to be the most effective method for producing biochar suitable for lead removal from aqueous solutions. The experimentally observed maximum adsorption capacity for the best-performing sample (AS-RAW-SP) was q_e_ = 103 ± 2.5 mg g^−1^ (*n* = 3; RSD = 2.4%), demonstrating the practical adsorption potential of slow pyrolysis almond shell biochar under the tested conditions. These findings contribute to the development of sustainable and efficient biochar-based remediation strategies using agricultural waste materials.

## Figures and Tables

**Table 1:** Proximate and elemental composition (wt.%).

Material	Moisture (wt.%)	Ash (wt.%)	Volatile Matter (wt.%)	Fixed Carbon (wt.%)	C (wt.%)	H (wt.%)	N (wt.%)	S (wt.%)
AS-RAW	0	3.1	79.4	17.4	45.60	6.40	1.74	0.30
AS-RAW-SP	0	2.9	8.3	88.8	87.86	0.96	0.78	n.a.
AS-KOH-SP	0	5.2	13.4	81.4	76.56	1.42	0.41	n.a.
AS-RAW-MW	0	2.0	8.5	89.5	87.93	1.10	0.96	n.a.
AS-KOH-MW	0	4.9	13.9	81.2	76.13	1.34	0.41	n.a.

Note: Values in Table 1 are reported on a dry-weight basis (moisture = 0). Original as-received moisture contents and the raw proximate data are available in Supplementary Materials Table S1. Where necessary, numbers in the text have been recalculated to a dry basis and this is stated explicitly. n.a.—not analyzed.

**Table 2:** Textural parameters of almond shell-derived biochars.

Material	SBET (m^2^·g^−1^)	V_net_ (cm^3^·g^−1^)	V_mic_ (cm^3^·g^−1^)	V_meso_ (cm^3^·g^−1^)	Median Micropore Size (nm)
AS-RAW-SP	693	0.598	0.161	0.437	0.6157
AS-KOH-SP	476	0.185	0.120	0.065	0.4766
AS-RAW-MW	147	0.144	0.037	0.107	0.5871
AS-KOH-MW	45	0.050	0.012	0.038	0.5945

## Data Availability

The original contributions presented in this study are included in the Supplementary Material. Further inquiries can be directed to the corresponding author.

## Supplementary Materials

The following supporting information can be downloaded at: https://www.mdpi.com/article/10.3390/molecules30204121/s1. Refs. [38,39,40,41,42,43,44,45,46,47,48,49,50,51,52,53,54] are cited in the Supplementary Materials.
