# Supplementary material for: Almond Shell-Derived Biochar for Lead Adsorption: Comparative Study of Pyrolysis Techniques and Sorption Capacities
Source: Molecules. 2025 Oct 17;30(20):4121. doi: 10.3390/molecules30204121 (PMC12565990; doi:10.3390/molecules30204121)
Supplement: Supplementary file 1 [file molecules-30-04121-s001.zip › molecules-3855403-supplementary.pdf]

Suplementary informations

Biochar characterisation

Proximate analysis of AS-RAW and AS-KOH samples was performed in accordance with ASTM D7582 [38] using a LECO TGA 701 thermogravimetric analyzer (LECO Corporation, St. Joseph, MI, USA). This method allows the determination of moisture, volatile matter, fixed carbon, and ash content, expressed as weight percentages (wt.%). The analysis followed a controlled temperature program enabling the sequential separation of each component. Moisture content was determined by low temperature drying. As the temperature increased, volatile matter was quantified. The remaining solid fraction was used to calculate fixed carbon by difference. Finally, the sample was combusted in oxygen at high temperature to determine the ash content. This analysis provided comprehensive data on the composition and thermal behavior of the materials.

Elemental analysis (CHNS) was conducted to determine the carbon, hydrogen, nitrogen, and sulfur content. The analysis employed high-temperature combustion (>1000 °C) in an oxygen atmosphere. Organic components were completely oxidized to CO_2, H_2O, SO_2, and NO_x. Nitrogen oxides were subsequently reduced in a reduction furnace, and the resulting gases were detected using a thermal conductivity detector (TCD). Helium (He) was used as the carrier gas. The measurements were performed using a LECO CNS628 analyzer (LECO Corporation, St. Joseph, MI, USA).

Thermogravimetric analysis (TGA) was used to assess the temperature-dependent mass changes of the raw (RAW) sample, providing insights into its thermal stability and suitability for pyrolysis. The analysis was performed using a STA 300 instrument (Hitachi, Japan). Samples were placed in α-Al_2O_3 crucibles and exposed to a nitrogen flow (100 $cm^3 \cdot min^{-1}$). Heating was applied at a rate of 10 $°C \cdot min^{-1}$ up to 800 °C.

The Brunauer–Emmett–Teller (BET) method was used to determine the specific surface area via nitrogen physisorption at cryogenic temperature. Prior to analysis, samples were degassed under vacuum (<1 Pa) at 350 °C for 48 hours. Adsorption measurements were carried out at −196 °C using a 3Flex analyzer (Micromeritics, USA). Surface area calculations were based on adsorption isotherms up to a relative pressure of $p/p_0 = 0.99$. The isotherms were interpreted using the IUPAC classification scheme for six types of adsorption isotherms[39,40].

Fourier-transform infrared spectroscopy (FTIR) was used to identify functional groups in the biochar. Spectra were recorded using a Nicolet iS50 FT-IR spectrometer (Thermo Fisher Scientific, Madison, WI,, USA) with the KBr pellet technique. Each spectrum was obtained as the average of 32 scans, with background subtraction and software-based correction using OMNIC (Eurolab, Prague, Czech Republic).

In our study, we analyzed lead using **atomic absorption spectrometry (AAS)**, working with liquid samples that we only filtered through a 25 μm pore size filter to remove unwanted solid particles prior to measurement. We analyzed these prepared samples using a contrAA700 atomic absorption spectrometer (Analytik Jena, Jena, Germany), which enabled us to measure using the flame technique (acetylene-air or acetylene-nitrous oxide) or with a graphite tube (using argon as an inert gas). The principle of our method was based on the absorption of primary radiation by lead atoms in the ground state, where we nebulized the sample and introduced the resulting aerosol into the flame, causing the solution to evaporate and chemical bonds to break. A light beam from a xenon lamp passed through the flame, with its photons being absorbed by lead atoms, resulting in a decrease in the intensity of the transmitted light, which we expressed as absorbance. For lead determination, we used the characteristic absorption line at 283.3 nm, and for low concentrations, we employed electrothermal atomization with detection limits in the ppb range.

Kinetic and Equilibrium Models

The pseudo-first-order kinetic model (Lagergren model) assumes that the adsorption rate is proportional to the difference between the equilibrium adsorption capacity and the amount of adsorbate at a given time [41]. This model is expressed by Equation S1 [42]

$$q_t = q_e(1 - e^{-k_1 t}) \quad \text{(S1)}$$

where:

q_t – amount of adsorbate at time t (mg·g⁻¹),

q_e – equilibrium adsorption capacity (mg·g⁻¹),

k_1 – pseudo-first-order rate constant (min⁻¹).

The pseudo-second-order model assumes that the rate-limiting step of the adsorption process may be chemisorption. This involves interactions between adsorbate and adsorbent based on electron sharing or exchange, resulting in stronger chemical bonding [43]. Compared to the pseudo-first-order model, it often provides a more accurate description of the adsorption mechanism [44].

The pseudo-second-order model is described by Equation S2 [42]:

$$q_t = \frac{(q_e{}^2 k_2 t)}{(1 + k_2 q_e t)} \quad \text{(S2)}$$

where:

k_2 – pseudo-second-order rate constant (g·mg⁻¹·min⁻¹).

All other variables are as previously defined.

The Elovich kinetic model is based on the assumption that during the adsorption process, the activation energy gradually increases, and the adsorbent surface is considered homogeneous [45] It is expressed by Equation S3 [42]:

$$q_t = \frac{1}{\beta} \ln(1 + \alpha\beta t) \quad \text{(S3)}$$

where:

α – initial adsorption rate (mg·g⁻¹·min⁻¹),

β – desorption constant (mg·g⁻¹).

All other variables are as previously defined.

The intra-particle diffusion (IPD) model (Weber–Morris) was used to evaluate the contribution of intra-particle diffusion to the overall adsorption process. The model is expressed as equation S4:

$$q_t = k_{id} t^{1/2} C \quad \text{(S4)}$$

where:

q_t – amount of Pb(VI) adsorbed at time t, (mg·g⁻¹),

t – contact time, (min),

k_{id} – intra-particle diffusion rate constant (slope of the linear fit), (mg·g⁻¹ min⁻¹ᐟ²),

C –intercept related to the thickness of the boundary layer, (mg·g⁻¹).

Equilibrium Tests

Adsorption isotherms are essential for understanding the interactions between adsorbent and adsorbate, and thus to optimize the efficiency of the adsorption process [46]. Equilibrium conditions were evaluated at initial Pb(II) concentrations of 50, 100, 200, 300, 400 and 500 mg·L⁻¹.

Although linear regression is commonly used for estimating model parameters, linearization can alter the dependent and independent variables, leading to error propagation. Therefore, nonlinear regression

was employed to improve the accuracy of parameter estimation, as it avoids the drawbacks associated with linearization. It has been shown to be a more reliable and effective method [47].

Four isotherm models were used for data fitting: Redlich–Peterson, Freundlich, Langmuir, and Dubinin–Radushkevich.

The Langmuir isotherm assumes monolayer adsorption onto a homogeneous surface with a finite number of identical sites and no interaction between adsorbed molecules. It is represented by Equation S5 [42]:

$$q_e = \frac{(q_{max} k_L c_e)}{(1 + k_L c_e)} \quad \text{(S5)}$$

where:

q_{max} – maximum adsorption capacity (mg·g⁻¹),

k_L – Langmuir constant (L·mg⁻¹),

c_e – equilibrium concentration of adsorbate (mg·L⁻¹).

All other variables are as previously defined.

The Freundlich isotherm is one of the earliest empirical models used to describe equilibrium adsorption data and to characterize adsorption on heterogeneous surfaces. It is described by Equation S6 [42]:

$$q_e = k_F c_e^{1/n} \quad \text{(S6)}$$

where:

k_F – Freundlich constant [(mg·g^{-1}) (L·mg^{-1})$^{1/n}$],

n – adsorption intensity.

All other variables are as previously defined.

The Redlich–Peterson isotherm is an empirical model used for adsorption on heterogeneous surfaces. It incorporates both Langmuir and Freundlich models and is expressed by Equation S7 [42]:

$$q_e = \frac{k_{RP} c_e}{1 + \alpha_{RP} c_e^g} \quad \text{(S7)}$$

where:

k_{RP} – Redlich–Peterson constants (L·g^{-1}),

α_{RP} – Redlich–Peterson constant (L mg^{-1}),

g – exponent between 0 and 1.

All other variables are as previously defined.

The Dubinin–Radushkevich isotherm was developed to consider the influence of porous structure on the adsorption process. It is represented by Equation S8 [42]:

$$q_e = q_{RD} e^{-k_{RD} \varepsilon^2} \quad \text{(S8)}$$

where:

q_{RD} – theoretical saturation capacity (mg·g^{-1}),

k_{RD} – constant related to sorption energy (mol^2·kJ^{-2}),

ε – Polanyi potential.

Polanyi potential calculation (see Equation S9):

$$\varepsilon = RT\ln(1 + \frac{1}{c_e}) \qquad \text{(S9)}$$

where R is the gas constant and T the temperature in Kelvin.

Effect of pH

The pH of the solution was adjusted using 1 M HNO_3 and 1 M NaOH within the range of pH 3–6. In all experiments, the pH was measured both before and after adsorption to assess the influence of biochar on solution chemistry. The pH was monitored using a pH meter (Hanna EDGE, Prague, Czech Republic).

Statistical Analysis and Model Fitting

To estimate the parameters of adsorption models, nonlinear regression analysis was employed. Unlike linearized forms of isotherm and kinetic models, which may alter the relationship between dependent and independent variables and propagate errors, nonlinear regression preserves the original mathematical structure and provides more accurate parameter estimation. This approach has been demonstrated to be a more reliable and effective tool for modeling adsorption processes [48].

All model fitting and statistical evaluations were performed using OriginPro 2023 (OriginLab Corporation, Northampton, MA, USA). Model parameters were estimated using nonlinear curves fitting with iterative optimization algorithms implemented in OriginPro. Goodness-of-fit was assessed using determination coefficients (R^2).

Suplementary figures

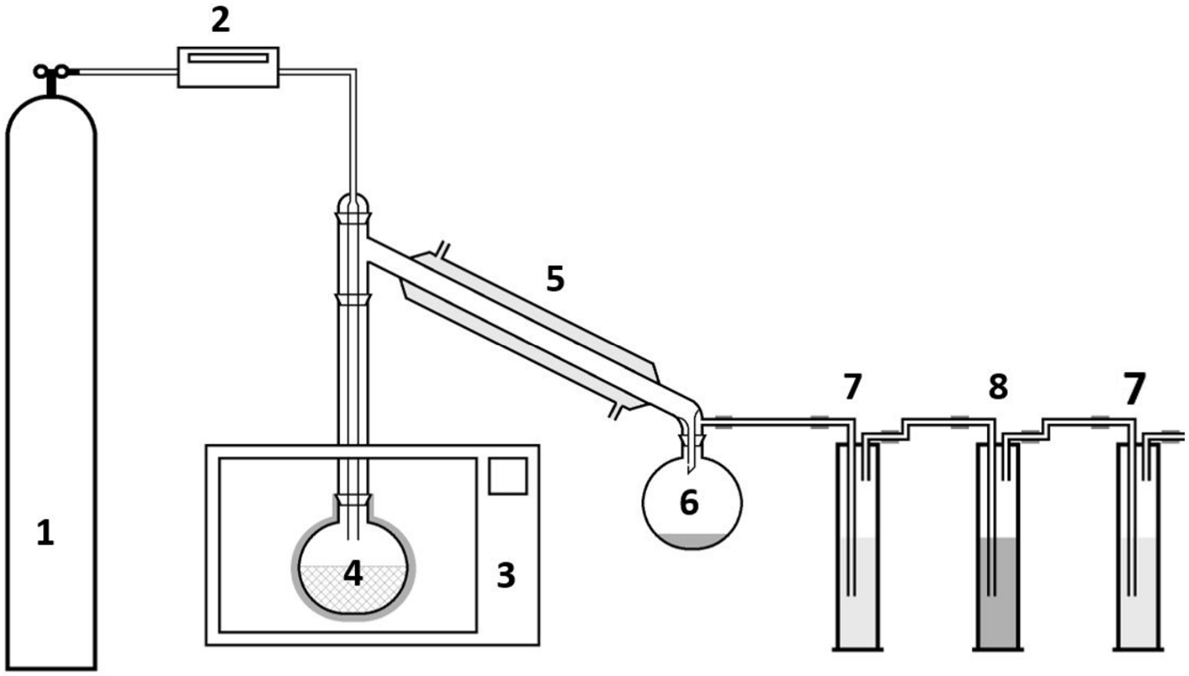

Figure S1: Diagram of microwave apparatus 1. Nitrogen pressure cylinder; 2. Nitrogen flow control; 3. Microwave oven; 4. Quartz reactor with a volume of 500 mL; 5. Cooler according to Liebig; 6. Condensation flask with a volume of 250 mL; 7. Washing flask with a volume of 250 mL with 70 mL of demineralized water; 8. Washing flask with a volume of 250 mL with 70 mL of Acetone (Penta, purity p.a)

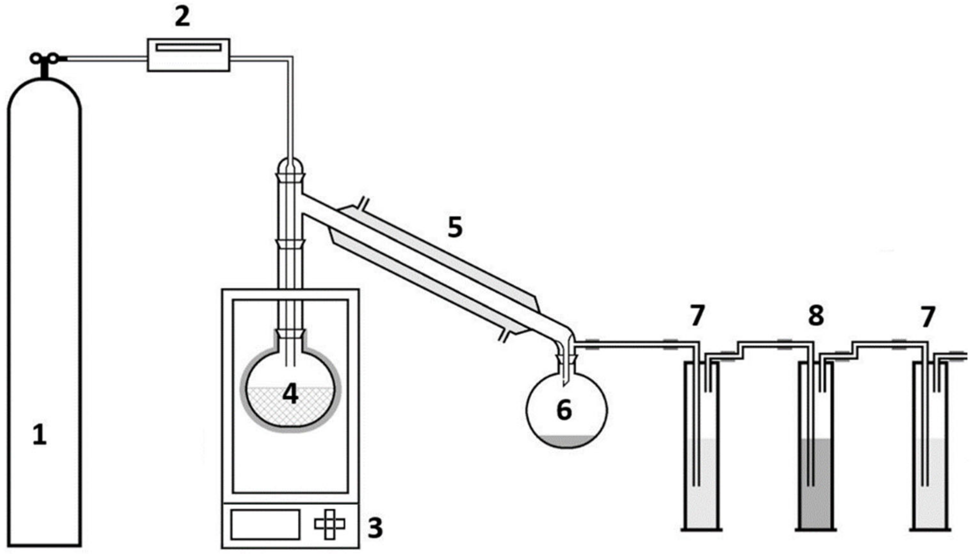

Figure S2: Schematic diagram of the apparatus for slow pyrolysis 1. Pressure bottle with nitrogen; 2. Nitrogen flow control; 3. Retort; 4. Quartz reactor with a volume of 500 mL; 5. Cooler according to Liebig; 6. Condensation flask with a volume of 250 mL; 7. Washing flask with a volume of 250 mL with 70 mL of demineralized water; 8. Washing flask with a volume of 250 mL with 70 mL of Acetone (Penta, purity p.a)

Table S1: Proximate and elemental composition (wt.%) with moisture.

Material	Moisture (wt.%)	Ash (wt.%)	Volatile matter (wt.%)	Fixed carbon (wt.%)	C (wt.%)	H (wt.%)	N (wt.%)	S (wt.%)
AS-RAW	9.10	2.83	72.20	15.86	45.60	6.40	1.74	0.30
AS-RAW-SP	6.42	2.70	7.75	83.14	87.86	0.96	0.78	n. a.
AS-KOH-SP	13.66	4.48	11.58	70.28	76.56	1.42	0.41	n. a.
AS-RAW-MW	6.15	1.89	7.94	84.03	87.93	1.10	0.96	n. a.
AS-KOH-MW	13.76	4.24	11.99	70.02	76.13	1.34	0.41	n. a.

Note: n. a. – not analyzed

Table S2: Reported Pb(II) adsorption capacities for agro-waste-derived biochars and comparison with this study

Feedstock / Treatment	Conditions (pH, C_0, m/V)	q_{max} or Removal (%)	Reference
**Almond shell biochar (slow pyrolysis)**	pH 4; C_0 = 50–500 mg·L⁻¹; m/V =2 g·L⁻¹	≈ 103 mg·g⁻¹ (this work)	This study
Pistachio shell biochar (powder)	pH 5; C_0 = 11.75 mg·L⁻¹; m/V = 0.8 g·L⁻¹	Removal ≈ 85.3 %	[49]
Pistachio shell biochar (high dose 10 g·L⁻¹)	C_0 = 15 mg·L⁻¹	Removal ≈ 100 %	[50]
Pistachio shell biochar (550 °C)	10 g·L⁻¹; C_0 = 15 mg·L⁻¹	≈ 100 % (Pb and Cu)	[51]
Corn cob	pH 5; C_0 =200 mg·L⁻¹; m/V =1 g·L⁻¹	87.08 mg·g⁻¹	[52]
Peanut shell biochar (pristine)	pH 5,5; C_0 =100 mg·L⁻¹; m/V =2 g·L⁻¹	56.5 mg·g⁻¹	[53]
Peanut & sheanut shells biochar (350 °C / 700 °C)	pH 5.98 to 7.38; C_0 =200 mg·L⁻¹; m/V =1 g·L⁻¹	98.2–100% removal (range)	[54]

Conditions are reported as given in the respective studies. Values of q_{max} are based on isotherm fitting where available, otherwise maximum reported removal is listed for comparability."

Table S3: Adsorption capacity values (q, mg·g⁻¹) obtained from triplicate experiments for each biochar and contact time, including mean, standard deviation (SD), and relative standard deviation (RSD).

Sample	Time (min)	q_1 (mg g⁻¹)	q_2 (mg g⁻¹)	q_3 (mg g⁻¹)	q_{Mean} (mg g⁻¹)	SD	RSD (%)
AS-KOH-MW	10	17.11	19.03	18.06	18.07	0.96	5.3
	20	16.10	15.10	16.05	16.05	0.05	0.3
	30	12.11	13.99	13.04	13.05	0.94	7.2
	45	14.33	14.12	14.24	14.23	0.10	0.7
	60	14.98	16.00	15.65	15.55	0.52	3.3
	120	16.16	16.57	16.11	16.28	0.25	1.6
	150	15.04	14.28	14.10	14.47	0.50	3.4
AS-RAW-MW	10	19.94	19.24	19.64	19.61	0.35	1.8

	20	19.62	19.02	19.22	19.29	0.31	1.6
	30	23.10	22.58	22.78	22.82	0.25	1.1
	45	18.05	18.99	18.55	18.53	0.48	2.6
	60	22.22	22.85	22.50	22.53	0.31	1.4
	120	18.13	19.01	18.67	18.60	0.44	2.4
	150	23.34	22.49	22.99	22.94	0.43	1.9
AS-KOH-SP	10	14.52	15.11	14.82	14.82	0.29	2.0
	20	13.15	14.45	13.74	13.78	0.65	4.7
	30	15.17	14.87	15.03	15.02	0.15	1.0
	45	16.11	15.56	15.86	15.84	0.28	1.7
	60	17.12	17.32	17.22	17.22	0.10	0.6
	120	16.18	15.38	15.79	15.78	0.40	2.5
	150	16.28	15.74	16.01	16.01	0.27	1.7
AS-RAW-SP	10	19.99	20.59	20.29	20.29	0.30	1.5
	20	23.42	23.00	23.21	23.21	0.21	0.9
	30	23.56	22.77	23.17	23.17	0.39	1.7
	45	16.49	15.65	16.08	16.10	0.42	2.6
	60	22.35	23.12	22.74	22.74	0.38	1.7
	120	22.81	23.67	23.23	23.24	0.43	1.9
	150	23.55	24.18	23.87	23.87	0.312	1.3

Table S4: Raw experimental adsorption values (q, mg·g^{-1}) measured at initial Pb(II) = 600 mg·L^{-1} for all biochars — value excluded from nonlinear model fitting.

**Sample**	**C_0 mg L^{-1}**	**q_1 mg g^{-1}**	**q_2 mg g^{-1}**	**q_3 mg g^{-1}**	**q_{mean} mg g^{-1}**	**SD**	**RSD %**
AS-RAW-SP	600	75.42	76.18	75.71	75.77	0.38	0.5
AS-RAW-MW		7.84	7.46	7.48	7.59	0.21	2.8

Note: The experimental adsorption values measured at initial Pb(II) = 600 mg·L^{-1} are reported here for completeness but were excluded from nonlinear isotherm fitting because they deviated markedly from the trend observed at lower concentrations.

In addition to the Elovich model considered here, the kinetic data were also fitted to the pseudo-first-order (PFO), pseudo-second-order (PSO), and intraparticle diffusion (IPD) models. Trial fits using Elovich, PFO, PSO, and IPD are provided for completeness (Figures S3–S6). However, due to the very rapid equilibration of Pb(II) adsorption, most data points already represent saturated conditions, which led to non-physical parameters (e.g., unrealistically high k_1 or negative k_2). Therefore, these models are not considered suitable for mechanistic interpretation in this study. Accordingly, parameters are not interpreted mechanistically and the fits serve only as descriptive summaries; the main text reports t_{90} and q_e.

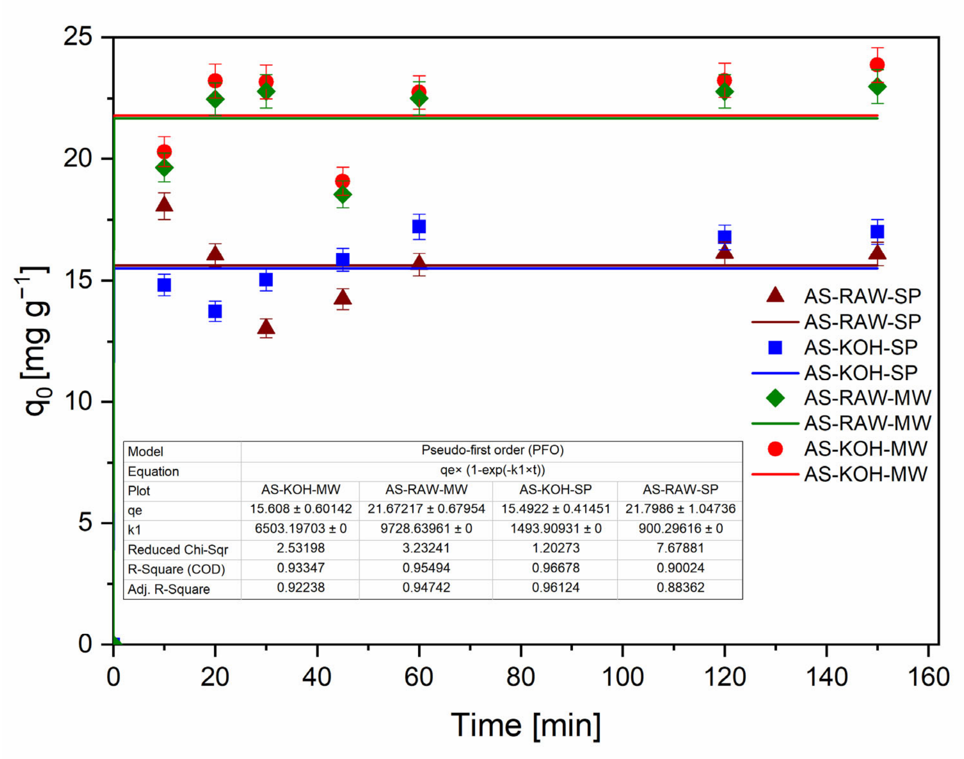

Model	Pseudo-first order (PFO)			
Equation	qe× (1-exp(-k1×t))			
Plot	AS-KOH-MW	AS-RAW-MW	AS-KOH-SP	AS-RAW-SP
qe	15.608 ± 0.60142	21.67217 ± 0.67954	15.4922 ± 0.41451	21.7986 ± 1.04736
k1	6503.19703 ± 0	9728.63961 ± 0	1493.90931 ± 0	900.29616 ± 0
Reduced Chi-Sqr	2.53198	3.23241	1.20273	7.67881
R-Square (COD)	0.93347	0.95494	0.96678	0.90024
Adj. R-Square	0.92238	0.94742	0.96124	0.88362

Figure S3: Pseudo-first order (PFO) kinetic model fitted to Pb(II) adsorption data for all biochars. Conditions: adsorbent dose = 0.1 g in 50 mL; initial concentrations 50 mg L^{-1}; contact time = 10, 20, 30, 45, 60, 120, and 150 minutes; t = ambient (22 ± 2 °C); shaker = 180 rpm. Fit method: nonlinear regression (OriginPro 2023).

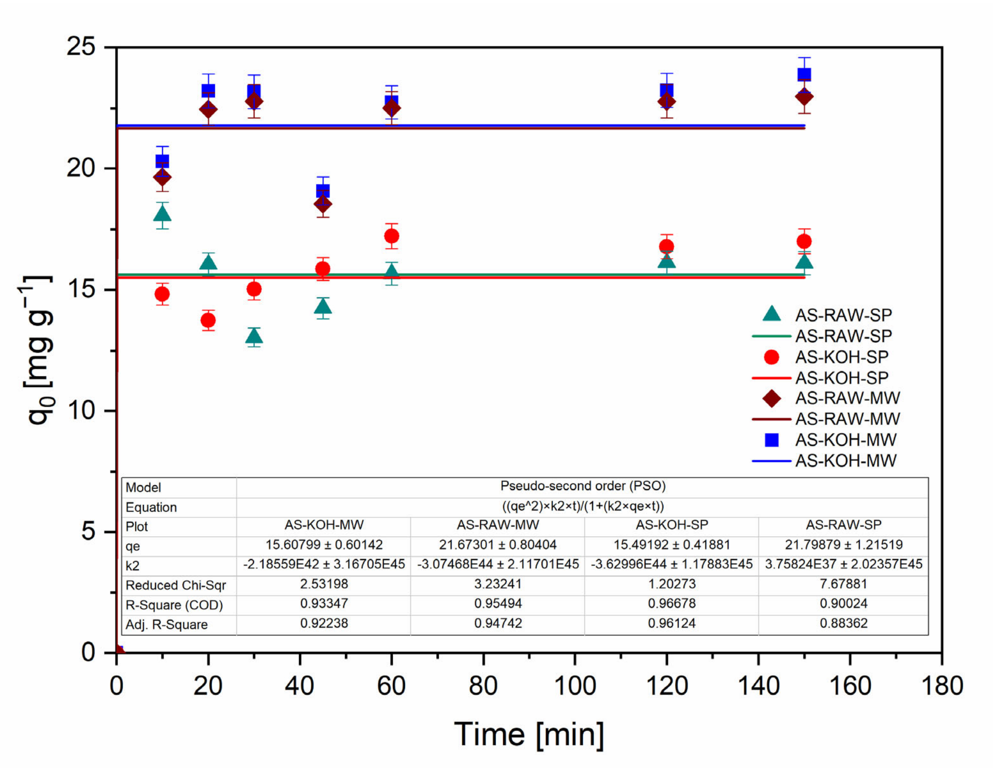

Model	Pseudo-second order (PSO)			
Equation	((qe^2)×k2×t)/(1+(k2×qe×t))			
Plot	AS-KOH-MW	AS-RAW-MW	AS-KOH-SP	AS-RAW-SP
qe	15.60799 ± 0.60142	21.67301 ± 0.80404	15.49192 ± 0.41881	21.79879 ± 1.21519
k2	-2.18559E42 ± 3.16705E45	-3.07468E44 ± 2.11701E45	-3.62996E44 ± 1.17883E45	3.75824E37 ± 2.02357E45
Reduced Chi-Sqr	2.53198	3.23241	1.20273	7.67881
R-Square (COD)	0.93347	0.95494	0.96678	0.90024
Adj. R-Square	0.92238	0.94742	0.96124	0.88362

Figure S4: Pseudo-second order (PSO) kinetic model fitted to Pb(II) adsorption data for all biochars. Conditions: adsorbent dose = 0.1 g in 50 mL; initial concentrations 50 mg L^{-1}; contact time = 10, 20, 30, 45, 60, 120, and 150 minutes; t = ambient (22 ± 2 °C); shaker = 180 rpm. Fit method: nonlinear regression (OriginPro 2023).

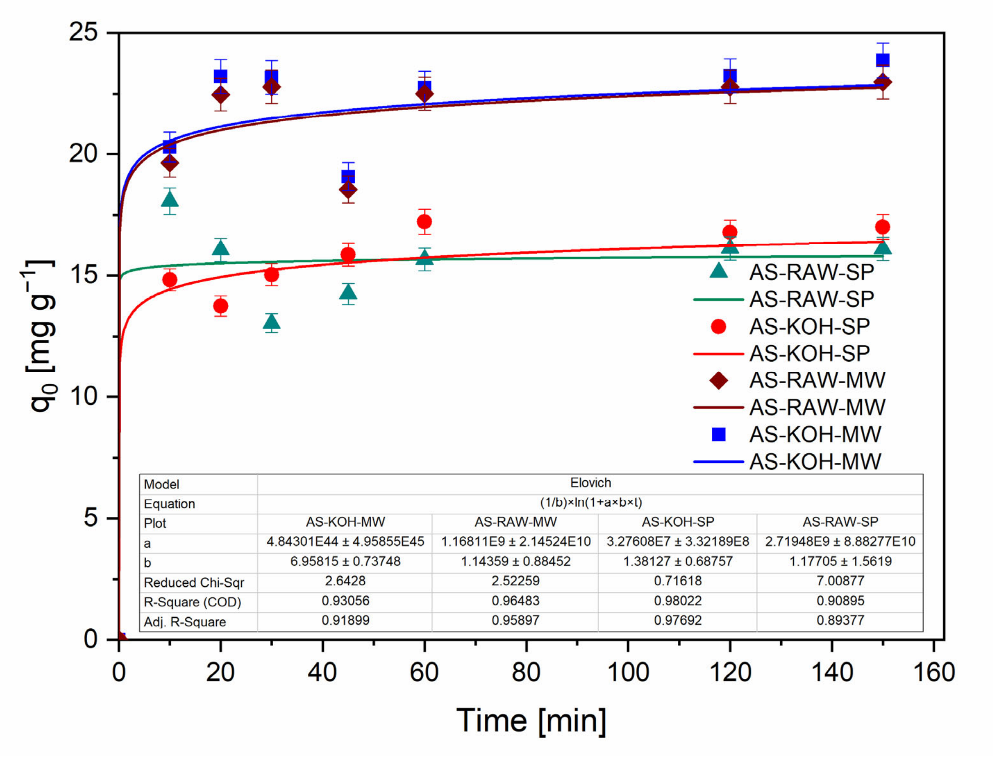

Model	Elovich			
Equation	(1/b)×ln(1+a×b×t)			
Plot	AS-KOH-MW	AS-RAW-MW	AS-KOH-SP	AS-RAW-SP
a	4.84301E44 ± 4.95855E45	1.16811E9 ± 2.14524E10	3.27608E7 ± 3.32189E8	2.71948E9 ± 8.88277E10
b	6.95815 ± 0.73748	1.14359 ± 0.88452	1.38127 ± 0.68757	1.17705 ± 1.5619
Reduced Chi-Sqr	2.6428	2.52259	0.71618	7.00877
R-Square (COD)	0.93056	0.96483	0.98022	0.90895
Adj. R-Square	0.91899	0.95897	0.97692	0.89377

Figure S5: Elovich kinetic model fitted to Pb(II) adsorption data for all biochars. Conditions: adsorbent dose = 0.1 g in 50 mL; initial concentrations 50 mg L^{-1}; contact time = 10, 20, 30, 45, 60, 120, and 150 minutes; t = ambient (22 ± 2 °C); shaker = 180 rpm. Fit method: nonlinear regression (OriginPro 2023).

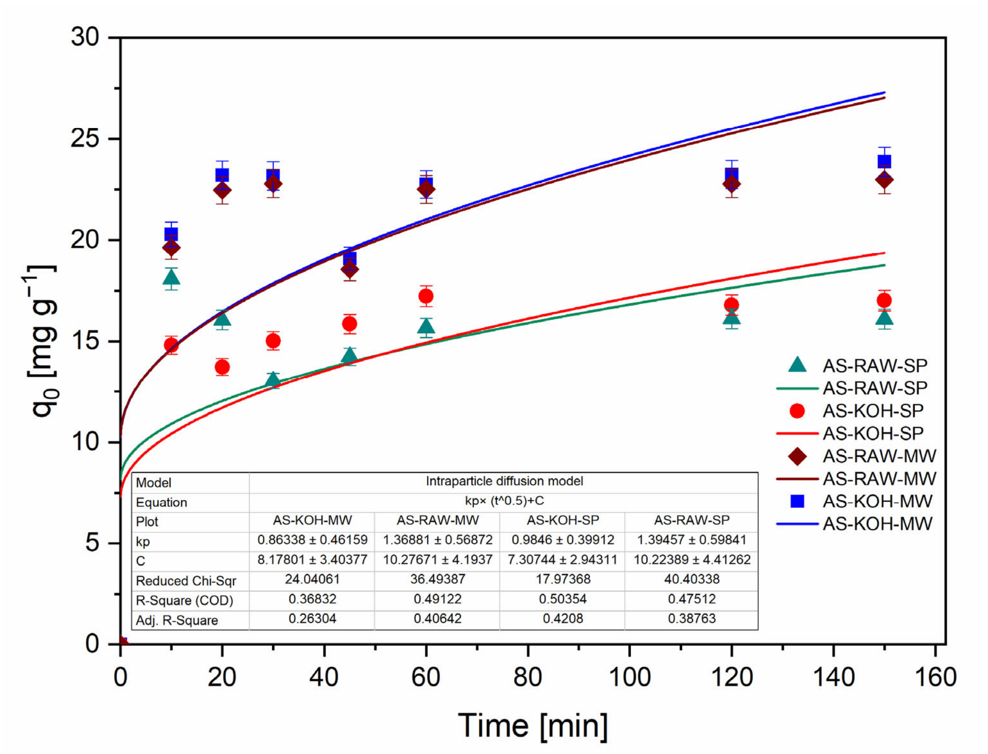

Model	Intraparticle diffusion model			
Equation	kp× (t^0.5)+C			
Plot	AS-KOH-MW	AS-RAW-MW	AS-KOH-SP	AS-RAW-SP
kp	0.86338 ± 0.46159	1.36881 ± 0.56872	0.9846 ± 0.39912	1.39457 ± 0.59841
C	8.17801 ± 3.40377	10.27671 ± 4.1937	7.30744 ± 2.94311	10.22389 ± 4.41262
Reduced Chi-Sqr	24.04061	36.49387	17.97368	40.40338
R-Square (COD)	0.36832	0.49122	0.50354	0.47512
Adj. R-Square	0.26304	0.40642	0.4208	0.38763

Figure S6: Intraparticle diffusion (IPD) model fitted to Pb(II) adsorption data for all biochars. Conditions: adsorbent dose = 0.1 g in 50 mL; initial concentrations 50 mg L^{-1}; contact time = 10, 20, 30, 45, 60, 120, and 150 minutes; t = ambient (22 ± 2 °C); shaker = 180 rpm. Fit method: nonlinear regression (OriginPro 2023).

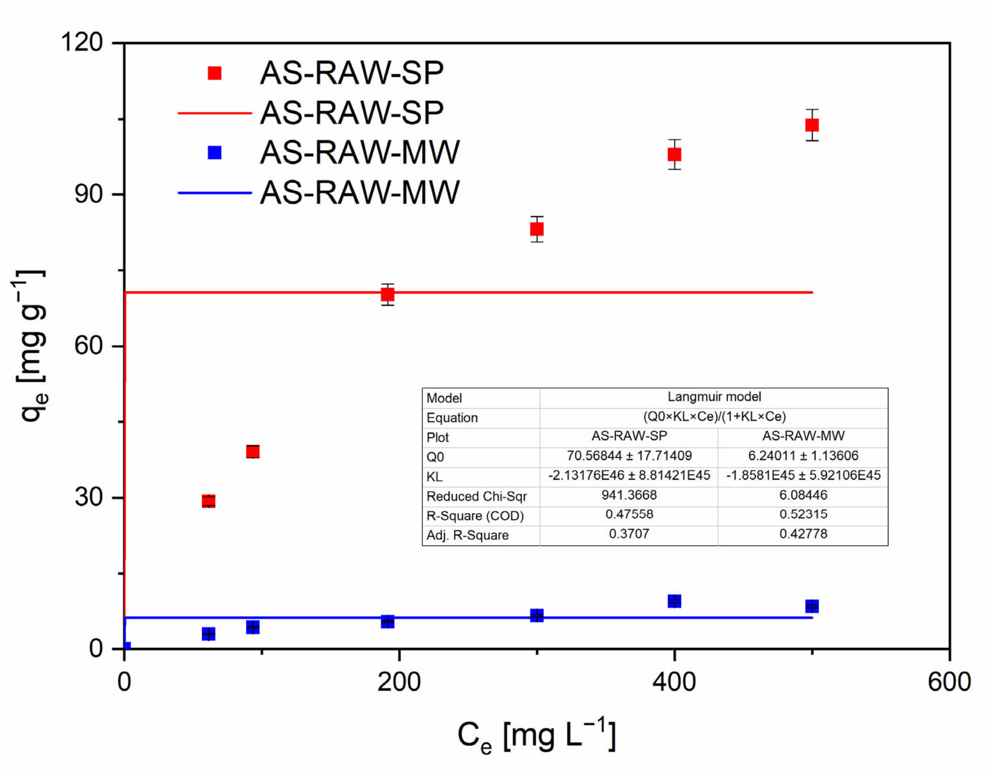

Model	Langmuir model	
Equation	(Q0×KL×Ce)/(1+KL×Ce)	
Plot	AS-RAW-SP	AS-RAW-MW
Q0	70.56844 ± 17.71409	6.24011 ± 1.13606
KL	-2.13176E46 ± 8.81421E45	-1.8581E45 ± 5.92106E45
Reduced Chi-Sqr	941.3668	6.08446
R-Square (COD)	0.47558	0.52315
Adj. R-Square	0.3707	0.42778

Figure S7: Langmuir adsorption isotherm for Pb(II) removal by almond shell biochars prepared by slow pyrolysis (AS-RAW-SP) and microwave pyrolysis (AS-RAW-MW). Experimental data points and model fits are shown; fitting parameters are provided within the figure. Conditions: adsorbent dose = 0.1 g in 50 mL, initial Pb(II) concentrations 50–500 mg·L^{-1}, equilibrium pH = 4 (adjusted with HNO_3/NaOH), contact time = 60 minutes, ambient t (22 ± 2 °C), n = 3 (mean); shaker = 180 rpm. Fit method: nonlinear regression (OriginPro 2023). The experimental value at 600 mg·L^{-1} is retained in Table S4 (SI) for completeness but was excluded from regression.

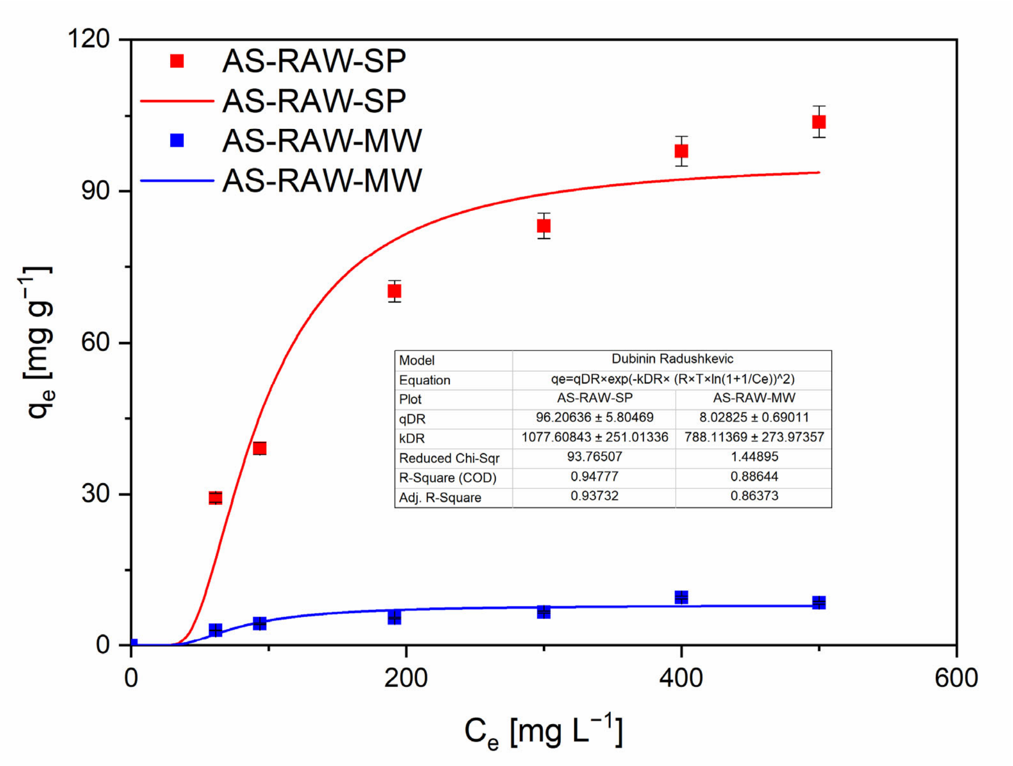

Figure S8: Dubinin Radushkevic adsorption isotherm for Pb(II) removal by almond shell biochars prepared by slow pyrolysis (AS-RAW-SP) and microwave pyrolysis (AS-RAW-MW). Experimental data points and model fits are shown; fitting parameters are provided within the figure. Conditions: adsorbent dose = 0.1 g in 50 mL, initial Pb(II) concentrations 50–500 mg·L^{-1}, equilibrium pH = 4 (adjusted with HNO_3/NaOH), contact time = 60 minutes, ambient t (22 ± 2 °C), *n* = 3 (mean); shaker = 180 rpm. Fit method: nonlinear regression (OriginPro 2023). The experimental value at 600 mg·L^{-1} is retained in Table S4 (SI) for completeness but was excluded from regression.
